# Primary spinal extradural hydatid cyst causing spinal cord compression

**DOI:** 10.4103/0019-5413.30531

**Published:** 2007

**Authors:** NN Gopal, SPS Chauhan, Nitin Yogesh

**Affiliations:** Neurosurgery Unit, Department of Surgery, M.L.N Medical College, Allahabad, India

**Keywords:** Hydatidosis, magnetic resonance imaging, spinal cord compression, surgery

## Abstract

Primary spinal hydatid disease is rare and represents an uncommon but significant manifestation of hydatid disease. We report a case of primary intraspinal extradural hydatid cyst of the thoracic region causing spinal cord compression. The presenting symptoms were mostly atypical and the diagnosis was established preoperatively on the basis of magnetic resonance imaging. The patient underwent surgery resulting in complete recovery and is recurrence-free after 24 months follow-up.

Hydatid disease of the spine is caused by the parasite *Echinococcus granulosus*, a helminth belonging to the cestode group. Hydatidosis of the bone occurs in 0.5-3% of all the cases: the vertebral column is involved in 50% of these.^1^,^2^ The disease usually spreads to the spine by direct extension from pulmonary or abdominal infestation and less often begins primarily in the vertebral body.^2^ Primary extradural hydatid disease without any systemic foci is extremely rare. It is a common cause of spinal cord compression in endemic areas and the diagnosis remains obscure until symptoms resulting from complications due to root and cord compression appear.^3^ Preoperative diagnosis is essential because the rupture and dissemination of cyst may result in anaphylaxis and recurrence.

We report a case of primary thoracic extradural hydatid cyst and review the literature.

## CASE REPORT

A 38-year-old man, farmer by occupation presented with gradually increasing back pain and progressive difficulty in walking since three months. Soon, he developed hesitancy of micturition which progressed to urinary incontinence. He also complained of numbness and altered sensations in both legs. General physical examination showed no abnormality. Neurological examination revealed spastic paraparesis and hypoaesthesia below T6 level. Power was reduced to Grade two in both the lower limbs and there was loss of sensations, especially to pain and fine touch. The superficial abdominal and cremastric reflexes were absent and plantars were extensor bilaterally. The knee and ankle jerk were exaggerated with bilateral ankle clonus.

**Figure 1:**
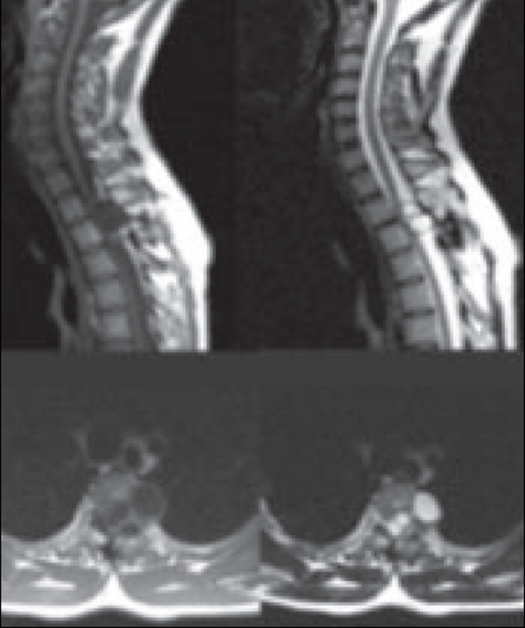
T1W- and T2W- sagittal (above) and axial (below) MRI images showing spinal cord compression

Plain X-ray of the thoracic spine did not reveal any abnormality. The magnetic resonance imaging (MRI) of thoracic spine revealed multiple well-defined extradural cystic lesions at T2-T3 level. There were cerebrospinal fluid (CSF) like signal intensities on T1- and T2- weighted images. [Figure 1]. No contrast enhancement was seen. Spinal cord was compressed anterolaterally. Ultrasonography of abdomen, chest X-ray and cranial CT were negative for any systemic foci. Serological test (ELISA) was also negative.

The patient underwent T1 to T4 laminectomy. Multiple pearly white cysts were found in the extradural space compressing the dural sac. All the cysts were extirpated without rupture. The operative field was soaked with hydrogen peroxide wetted patties for a few minutes and then washed with normal saline. Histopathological examination confirmed the diagnosis of hydatid cyst. Postoperative period was uneventful. Albendazole was given for a period of three months. There was complete regain of sensation in two to three weeks, although motor functions and bladder control recovered gradually over six weeks. There was no recurrence during the follow-up period of two years.

## DISCUSSION

Echinococcosis affecting the spine was first described by *Churrier* in 1807.^4^ The disease has a characteristic geographic distribution and is prevalent in most of the sheep-raising regions of the world.^1^,^3^ The infestation occurs either by direct ingestion of parasitic eggs from contact with dogs or indirectly from contaminated water or food.^2^ The cysts are transported from the intestinal wall via the blood stream to different organs. As the liver and lungs are the major filters of the body, they are most commonly located in the liver (60-70%) and lungs (10-15%); involvement of other organs is rare.

Vertebral hydatidosis occurs mostly between 30-50 years of age. A few cases have been reported in children also. The sites affected are the thoracic spine in 50%, lumbar spine in 20% and sacral spine in 20%.^4^ Cervical involvement is infrequent. The disease usually begins in the vertebral body preferentially in the center of the vertebra. There is a predilection for involvement of the pedicle. The intervertebral disc is usually spared. Perforation of the cortex and periosteum results in extraosseous extension which may be extraspinal or intraspinal.^5^ Primary extradural hydatid cyst is rare; primary intramedullary disease extremely rare.^5^,^6^ Primary extradural hydatid disease of spine can be explained through direct porto-vertebral venous shunts.^7^,^8^ The cysts are located epidurally and can be single or multiple. The primary cyst contains daughter cyst and microruptures can give rise to secondary cysts.

Clinically, spinal echinococcosis manifests by radicular pain associated with objective sensory and motor disturbances and local tenderness at the level of the involved vertebra. There are no pathognomic signs and symptoms of this disease. For this reason it is often misdiagnosed initially as tuberculosis of the spine, spinal tumor or disc herniation.

Although CT efficiently demonstrates bony erosion and the extent of the lesion, MR can demonstrate any cord compression throughout the length of the spinal cord and thus is the investigation of choice.^5^ On MRI, hydatid cysts appear as well-circumscribed, cystic lesions, with CSF-like signal intensities. The cyst wall is usually thin and regular with no septations. The cysts are hypointense on T1W images.^9^ On T2W images they appear hyperintense with sharply defined, hypointense cyst wall which shows mild enhancement following intravenous gadolinium, reflecting the vascularity of the pericyst.^10^ There is no contrast enhancement seen after intravenous gadolinium-enhanced MRI either in extradural or intradural hydatid cysts.^11^ Extradural spread of hydatid cysts through widened neural foramina into the muscle planes may result in a “bunch of grapes” appearance.^9^ Jena *et al*^12^ pointed out that the intensity differences in T2W sequences of MRI can also determine the viability of the cyst.

The treatment is essentially surgical and decompressive laminectomy with total excision of cyst, whenever possible, represents the treatment of choice.^5^ However, the invasive nature of the infestation in the spine precludes total removal and therefore permanent eradication.^1^,^13^ Spinal hydatidosis has been compared to malignant disease of spine (*le cancer blanc*).^8^,^13^ However, a prolonged and acceptable life still results following surgery. A recurrence risk of 30-40% has been described.^13^ There is a correlation between cyst localization and recurrence. To minimize the risk of recurrence, peroperative use of scolicidal agents like hypertonic saline, 10% formaldehyde, 0.5% silver nitrate and povidone iodine has been advocated. Postoperative chemotherapy with mebendazole or albendazole has been recommended for a period of at least three months.^3^,^7^ Surgery may have to be repeated several times to eradicate the disease completely. Pamir *et al*^1^ reported that 30% of their patients had previously undergone surgery.

Primary spinal echinococcosis must be considered in the preoperative differential diagnosis of the atypical presentation of vertebral lesions, especially in patients with risk factors. Early diagnosis and sugery combined with antihelminthic therapy of sufficient duration are mandatory to at least halt the progression of symptoms, but these measures do not provide a lasting solution.^14^–^16^

## References

[1] Pamir MN, Akalan N, Ozgen T, Erbengi A (1984). Spinal hydatid cysts. Surg Neurol.

[2] Charles RW, Govender S, Naido KS (1988). Echinococcal infection of the spine with neural involvement. Spine.

[3] Morris DL, Richards KS (1992). Hydatid disease. Current Medical and Surgical Management.

[4] Rayport M, Wisoff HS, Zaiman H (1964). Vertebral echinococcosis: Report of case of surgical and biological therapy with review of the literature. J Neurosurg.

[5] Abbasioun K, Amirjamshadi A (2001). Diagnosis and management of hydatid cyst of CNS: Hydatid cysts of skull orbit and spine. Neurosurgery.

[6] Ley A, Marti A (1970). Intramedullary hydatid cyst. Case report. J Neurosurg.

[7] Fiennes AG, Thomas DG (1982). Combined medical and surgical treatment of spinal hydatid disease. J Neurol Neurosurg Psychiatry.

[8] Morshed AA (1977). Hydatid disease of spine. Neurochirurgia.

[9] Tekkok IH, Benli K (1993). Primary spinal extradural hydatid disease: Report of a case with magnetic resonance characteristics and pathological correlation. Neurosurgery.

[10] Gupta S, Rathi V, Bhargava SK (2002). Ventricular primary spinal extradural hydatid cyst - MR appearance. Indian J Radiol Imaging.

[11] Hilmani S, El Malki M, Bertal A, Achowi M, Sami A, Onbouknlik A (2004). Lumbar intradural hydatid cyst - Case report. Neurochirurgie.

[12] Jena A, Tripathi RP, Jain AK (1991). Primary spinal echinococcosis causing paraplegia- Case report with MR and pathological correlation. AJNR.

[13] Turtas S, Sehrbundt V, Pau A (1980). Long term Results of surgery for hydatid disease of the spine. Surg Neurol.

[14] Ozdemir HM, Ogun TC, Tasbas B (2004). A lasting solution is hard to achieve in primary hydatid disease of the spine - Long term results and an overview. Spine.

[15] Sapkas GS, Machini TG, Chloros GD, Fountas KN, Themistocleous GS, Vrettakos G (2006). Spinal hydatid disease - A rare but existent pathological entity - A case report and review of literature. South Med J.

[16] Sharma NK, Chitkara N, Bakshi N, Guupta P (2003). Primary spinal extradural hydatid cyst. Neurol India.

